# Comparison of Population-Weighted Exposure Estimates of Air Pollutants Based on Multiple Geostatistical Models in Beijing, China

**DOI:** 10.3390/toxics12030197

**Published:** 2024-03-01

**Authors:** Yinghan Wu, Jia Xu, Ziqi Liu, Bin Han, Wen Yang, Zhipeng Bai

**Affiliations:** 1 State Key Laboratory of Environmental Criteria and Risk Assessment, Chinese Research Academy of Environmental Sciences, Beijing 100012, China; wuyinghan21@mails.ucas.ac.cn (Y.W.); ziqi@uw.edu (Z.L.); hanbin@craes.org.cn (B.H.); baizp@craes.org.cn (Z.B.); 2 Department of Environmental & Occupational Health Sciences, School of Public Health, University of Washington, Seattle, WA 98105, USA

**Keywords:** PM_2.5_, NO_2_, geostatistical modeling approach, exposure estimates, misclassification

## Abstract

Various geostatistical models have been used in epidemiological research to evaluate ambient air pollutant exposures at a fine spatial scale. Few studies have investigated the performance of different exposure models on population-weighted exposure estimates and the resulting potential misclassification across various modeling approaches. This study developed spatial models for NO_2_ and PM_2.5_ and conducted exposure assessment in Beijing, China. It explored three spatial modeling approaches: variable dimension reduction, machine learning, and conventional linear regression. It compared their model performance by cross-validation (CV) and population-weighted exposure estimates. Specifically, partial least square (PLS) regression, random forests (RF), and supervised linear regression (SLR) models were developed based on an ordinary kriging (OK) framework for NO_2_ and PM_2.5_ in Beijing, China. The mean squared error-based R^2^ (R^2^_mse_) and root mean squared error (RMSE) in leave-one site-out cross-validation (LOOCV) were used to evaluate model performance. These models were used to predict the ambient exposure levels in the urban area and to estimate the misclassification of population-weighted exposure estimates in quartiles between them. The results showed that the PLS-OK models for NO_2_ and PM_2.5_, with the LOOCV R^2^_mse_ of 0.82 and 0.81, respectively, outperformed the other models. The population-weighted exposure to NO_2_ estimated by the PLS-OK and RF-OK models exhibited the lowest misclassification in quartiles. For PM_2.5_, the estimates of potential misclassification were comparable across the three models. It indicated that the exposure misclassification made by choosing different modeling approaches should be carefully considered, and the resulting bias needs to be evaluated in epidemiological studies.

## 1. Introduction

Long-term exposure to air pollutants has been proven to be associated with adverse health outcomes [1,2,3]. Current research has focused on a medium- and long-term period exposure assessment at an individual level [4], which needs accurate exposure assessment at a fine spatial scale to eliminate exposure errors [5,6]. It is a big challenge because of the sparse monitoring stations and missing coverage of specific predictors, such as satellite-based models [7].

Geostatistical models developed by the land-use regression (LUR) approach have been widely used to assess medium- and long-term exposure to air pollution in epidemiological studies [8,9]. The geostatistical models were created based on the inputs of observational and geographic data sets, in which the former was involved as outcome variables and the latter were predictor variables. The choice of model development approaches, the method for dealing with predictor variables, and the model structures can all affect model performances [10,11,12,13]. The golden standard method for model evaluation includes out-of-sample validation and hold-out cross-validation (CV) [10,14]. However, some studies laced sufficient observational data for out-of-sample model validation or hold-out CV. Also, some results for evaluating some two-step models were not easy to interpret, e.g., the predictor selection and dimension reduction methods [15,16]. In brief, evaluating the performance of exposure models developed based on limited monitoring sites was challenging, but they are still helpful for environmental health studies. In previous studies on modeling approach comparison [10,17], the model performance was evaluated by cross-validation or out-of-sample validation. Nevertheless, to further compare these models’ performances, they need to be evaluated by exposure assessment in the real world, and potential exposure bias caused by different modeling algorithms needs to be assessed.

In this study, we used three modeling approaches, variable dimensionality reduction, machine learning, and variable screening, to build geostatistical models for NO_2_ and PM_2.5_ and to compare their model performance by estimating the misclassification of population-weighted exposure estimates in quartiles.

## 2. Methods

### 2.1. Study Area and Observations at Monitoring Sites

**Figure 1:**
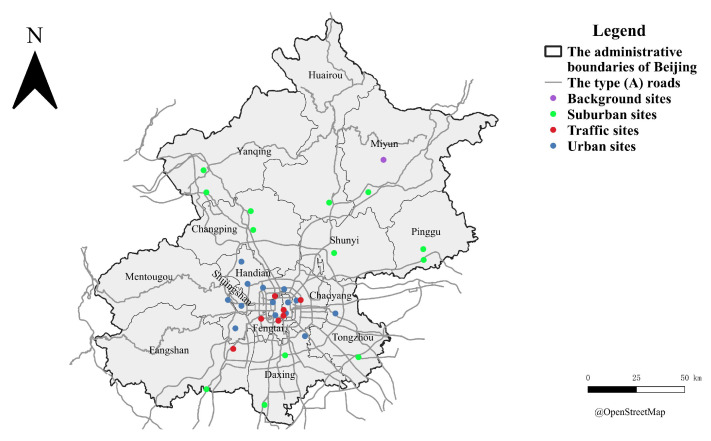
Map of the 35 monitoring sites in Beijing.

Beijing, China’s capital city, is a mega-city with a population of 21.5 million during the research period (2015–2020) [18]. Ambient concentrations of PM_2.5_ have decreased since stringent national and local environmental regulations were implemented in 2014 [19]. However, the ambient exposure level of such air pollutants is still higher than the World Health Organization (WHO) Global Air Quality Guidelines, 10 μg/m^3^ for NO_2_ and 5 μg/m^3^ for PM_2.5_ [20]. This study obtained NO_2_ and PM_2.5_ observational data from the Beijing air quality monitoring network (Beijing Municipal Environmental Monitoring Center). The NO_2_ was measured using the ultraviolet fluorescence method, and the PM_2.5_ was measured using the micro oscillating balance method at these monitoring sites [21]. A total of 35 monitoring sites were divided into 4 types, including 1 background site, 7 traffic sites, 14 urban sites, and 13 suburban sites, as shown in Figure 1. The background site is located near a reservoir in the Miyun district. The lowest NO_2_ and PM_2.5_ concentrations were observed at this background site. The traffic sites are close to the (A) type roads (highways and arterial roads) with a distance of less than 250 m. The other 27 monitoring sites were divided into urban and suburban sites, in which the urban sites were located inside and around the 6th-ring road, and the suburban sites were the rest of them.

**Figure 2:**
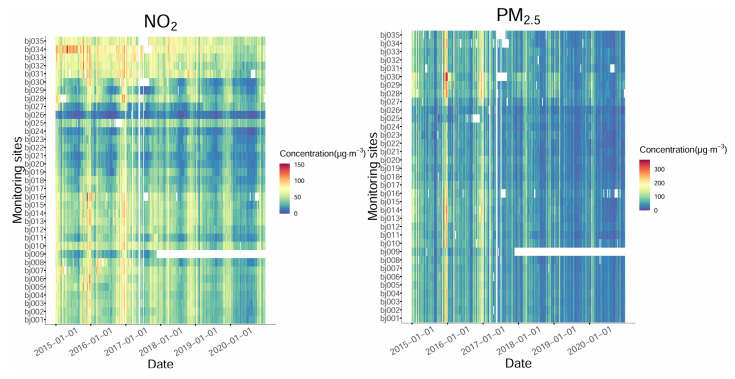
Weekly average concentrations of NO_2_ and PM_2.5_ at the 35 monitoring sites in Beijing from 2015 to 2020.

Annual average concentrations were obtained from the raw hourly concentrations. First, the daily averages were calculated by hourly concentration, following a criterion that 50% of the data (12 h) were available at each monitoring site. Second, these daily averages were used for calculating weekly averages, following the same criterion that 3-day data were available. Third, these weekly averages were applied for calculating annual averages, following another criterion of 25%. It allows the yearly averages to be calculated based on daily, weekly, and seasonal bases and be temporally representative. A similar temporal average strategy was used in Araki et al.’s NO_2_ exposure modeling study [22]. Figure 2 depicts the calculated weekly average concentrations of NO_2_ and PM_2.5_, where blank spaces represent missing concentrations. The above criteria screened some of the missing data, and the rest were due to the raw data loss. Table 1 shows the statistical summary of annual average NO_2_ and PM_2.5_ concentrations. In 2016 and 2017, the available yearly averages were less than 35 because of the data screening criteria. After 2018, one of the 35 monitoring sites, the Beijing Botanical Garden site, was excluded from the monitoring network, as shown in Figure 1.

### 2.2. Geographic Variables

In this study, we collected a wide array of geographic variables, including population density, traffic network, features (e.g., airport, rail yard, railways, etc.), points of interest (POI), land-use types, Normalized Difference Vegetation Index (NDVI), topography and coordinate variables. Some of these geographic variables were related to the emission sources, such as road network, features, and POI, while others were derived from the natural characteristics in the study area, e.g., population density and elevation. The details of these geographic variables are described in Table S1 in the Supplementary Information (SI).

### 2.3. Algorithms for Model Development

In this study, three types of modeling approaches were applied for model development. First, we chose the partial least squares (PLS) to represent the dimension reduction modeling approach. Second, random forest (RF), an advanced and relatively simple modeling approach with relatively fewer steps, was selected as a machine learning algorithm. Third, the supervised linear regression (SLR) algorithm was chosen as a traditional modeling approach. The PLS and SLR algorithms are based on a linear regression framework [10,16]. The RF algorithm is a complex algorithm dealing with non-linear relationships between response and predictor variables and also between predictor variables [10]. The PLS and SLR models have clear and interpretable frameworks, while the RF model is less interpretable because of its hidden black box [23].

Specifically, the PLS regression algorithm reduces predictor variables to a smaller set of uncorrelated components and performs least squares regression on these components. The PLS decomposes the large geographic variable matrix into a sequence of orthogonal PLS scores computed to maximize the covariance between concentrations and their prediction. According to the experience in our previous study in Beijing [11,24], the number of PLS scores was set to be 3.

As a machine learning algorithm, RF is an ensemble learning method that combines multiple decision trees to improve the accuracy and robustness of the developed models [23]. In RF model development, potential predictor variables are forced to be partitioned into subsets, which include separate decision trees for training. The output is the average of the decision tree simulation results. The RF model provides an importance evaluation index of the variables (IncMSE) that can be used to determine the influence of predictor variables on the response variables [25]. In this study, the setting of the RF models was based on our previous experience [11]. The coefficients of the random sampling times (mtry), number of decision trees in a random forest (ntree), and a minimum number of decision tree nodes (node size) were set to be 50, 500, and 5, respectively.

The SLR model is a traditional and widely used stepwise linear regression method developed using selected geographic variables as predictors [26,27]. First, univariate linear regression is conducted to find a starting point for an SLR model, as the highest R^2^ is obtained. Second, the additional predictor is added in each round to obtain the most significant increase in R^2^ until the rise of the R^2^ is less than 0.1. The selected variable in each round is available when its direction is plausible with the outcome pollutant. Third, the variance inflation factor (VIF) is applied to prevent multicollinearity. The selected variables with a value of VIF more than 3 were removed [28].

All the models were developed by R software (R 4.2.0, https://www.r-project.org/, accessed on 1 April 2022), using the R packages of “pls”, “randomForest” and so on.

### 2.4. Model Structure and Validation

For LUR models, their residuals are always spatially correlated [12]. Thus, we applied a two-step model structure, a LUR model with ordinary kriging (OK), to develop a LUR-OK model. First, a LUR model was developed; second, the residuals of the LUR model were further explained by OK. The same or similar model structures were widely used in previous studies [29,30,31].

The developed models were evaluated by using leave-one-site-out cross-validation (LOOCV). The observed data were split into groups equal to the number of monitoring sites, in which each group included the observations from one monitoring site. One data group was used for testing (testing group), and the other remaining data groups (training group) were used to fit the model. Then, the fitted model was used to predict the testing group and repeated until predictions for all groups were generated. We used mean square error based-R-Squared (*R*^2^_mse_), regression-based *R*^2^ (*R*^2^_reg_), and root-mean-square error (*RMSE*) to assess the accuracy and prediction ability of the model, which was computed on observations ($y_{i}$) and predictions (${\hat{y}}_{i}$) according to the equations below:

(1) $$RMSE=\sqrt{\frac{1}{n}\sum_{i=1}^{n}\left(y_{i}-{\hat{y}}_{i}\right)}$$

(2) $${R^{2}}_{mse}=\max\;(0,1-\frac{{RMSE}^{2}}{\sum_{i=1}^{n}{\left(y_{i}-\bar{y}\right)}^{2}/n})$$

In both equations, *n* is the number of observations, and $\bar{y}$ is the mean of observations. *R*^2^_mse_ is a measure of fit to the 1:1 line and is typically lower than *R*^2^_reg,_ which is a measure of fit to the regression line. The model performance was mainly evaluated by *R*^2^_mse_ and *RMSE*, and the *R*^2^_reg_ was also reported for comparisons with other studies.

## 3. Results and Discussion

### 3.1. Model Development

#### 3.1.1. PLS Models

**Figure 3:**
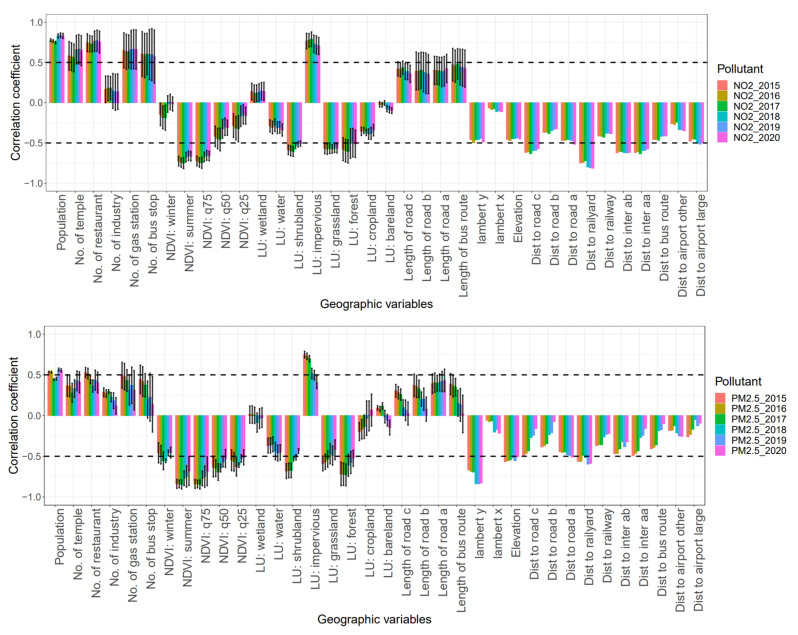
Correlation coefficients between the first PLS score and the geographic variables. Each box denotes the mean value of the correlation coefficient with corresponding geographic variables across the buffers, and the upper and lower bars represent the standard deviation across the buffers. The abbreviations of each geographic variable are shown in Table S1 in Supplementary Information.

The geographic variables were downscaled using the PLS approach. The first three PLS scores were obtained as inputs for the PLS models. The first PLS score explains most variations across the geographic data set. Thus, we evaluated correlation coefficients between the first PLS score and geographic variables to represent the influence of the geographic variables on PLS models. Figure 3 depicts these correlation coefficients for the annual NO_2_ and PM_2.5_ models. For NO_2_ models, the correlation coefficients between the first PLS score and the geographic variables were relatively stable across the annual models. The variables of all the annual NO_2_ models that were highly correlated with the first PLS score were population density, POI variables (the count of temples, restaurants, gas stations and bus stops), NDVI variables (NDVI in summer and the 75th percentile of NDVI), land-use type variables (shrubland, impervious, grassland, and forest), and the proximity variables (the distance to the type (C) road, railway, the intersection between type (A) and type (B) roads, and the intersection between type (A) roads.

Compared with the annual NO_2_ models, the correlation coefficients between the first PLS score and the geographic variables varied among the annual PM_2.5_ models, especially for the annual PM_2.5_ models after 2017. The variables of all the annual PM_2.5_ models with an absolute value of these correlation coefficients greater than 0.5 included the NDVI variables (NDVI in summer and the 75th, 50th, and 25th percentiles of NDVI), land-use type of forest, longitude (Lambert y), elevation, and distance to the railyard. The contrast of the correlation coefficients between these models presented different emission sources of NO_2_ and PM_2.5_. As a traffic-related primary air pollutant, the spatial distribution of NO_2_ was correlated with the geographic variables of road networks. In contrast, PM_2.5_ was correlated with the longitude variable because of its partially secondary species formation that originated from long-distance transportation [32].

#### 3.1.2. RF Models

**Figure 4:**
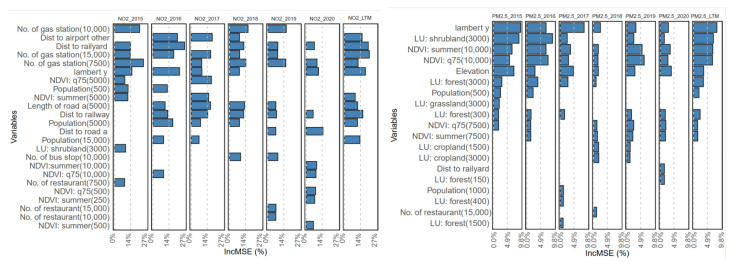
The geographic variables with the top ten IncMSE values in the RF model results.

According to the IncMSE of the variables given by the RF models, the top ten variables were summarized in Figure 4. The traffic-related variables significantly influence the NO_2_ models. The variables of distance to the railyard, the count of gas stations, the distance to the type (A) road, and the sum of the type (A) road length had relatively high IncMSE values in all NO_2_ models. For PM_2.5_, the most important variables were longitude, shrubland, and NDVI. Similar variable sensitivities were observed in the PLS models for PM_2.5_, in which the longitude variable was also highlighted.

#### 3.1.3. SLR Models

**Figure 5:**
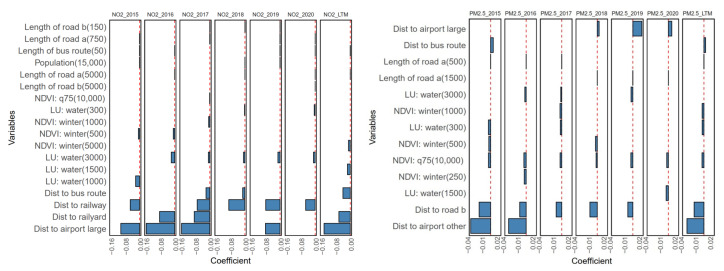
The coefficients of the variables selected by the SLR model.

Figure 5 shows the selected geographic variables by the SLR model and their regression coefficients. For NO_2_ models, the traffic-related variables were involved in model development. It is consistent with the RF models. Specifically, the coefficients of the variables of the distance to the airport, railway, and railyard had relatively high values. In addition, the NDVI and land-use type of water variables were also selected. Regarding PM_2.5_, the annual PM_2.5_ models selected fewer variables than the NO_2_ models. The variables of the distance to the airport and the distance to the type (B) road influenced the annual PM_2.5_ models a lot.

#### 3.1.4. Models Developed in the Urban Area

In order to compare model performance between the geostatistical models developed in the whole Beijing area and inside the 6th-ring road area that covered the total urban area. The LUR–urban (LURU) models were developed based on the monitoring sites inside and around the 6th-ring road. The coefficients of these LURU models are shown in Figures S1–S3.

For the urban model developed by the PLS approach (PLSU), some variables’ correlation coefficients had significant changes, such as the variables of the count of industries (POI), water land-use type, latitude, and distance to airports for the NO_2_ models and the variables of the count of industry (POI), NDVI in winter, latitude, and distance to the airports for PM_2.5_ models. The predictors in the PLS and PLSU models were different. It indicated that the PLS approach could be affected when the range of the observational data was expanded as the suburban areas were included. Different model coefficients were also found when comparing RF and RFU models, SLR and SLRU models.

### 3.2. Model Performance

The LOOCV results of NO_2_ and PM_2.5_ models are shown in Table 2 and Table 3, respectively. The performances of the LUR and LUR-OK models were summarized in each table. Generally, the annual PLS models performed better than others, with a higher range of R^2^_mse_ for NO_2_ (0.72~0.82) and PM_2.5_ (0.80~0.89), which were higher than the previous studies in Beijing [33,34]. Regarding the LTM of NO_2_, the PLS models had comparable good performance with the SLR models, which performed better than the RF models. Compared with the NO_2_ models without an OK in the model structure, the LUR-OK models improved the model performance for PLS and SLR LTM models with a slightly higher R^2^_mse_. For annual NO_2_ models, some performed worse when adding OK to the model structure. However, adding OK into the model structure worked well for RF models of PM_2.5_. For annual and LTM PM_2.5_, the RF-OK models improved their performance with an increase of 0.06~0.21 in R^2^_mse_ compared with the RF models. In comparison, these increases in R^2^_mse_ of PLS and SLR models because of adding OK were −0.17~0.01 and −0.09~0.08, respectively. For the LTM of PM_2.5_, the PLS-OK model performed best among the PLS, RF, and SLR models with or without OK. A spatiotemporal covariate model was built in a previous study with an R^2^_mse_ of 0.93, and its RMSE was 1.72 μg/m^3^ [35]. Improved performance by adding OK in the model structure was also found in a previous study [36]. The good performance of the PLS approach on spatial modeling was concluded in the previous study [11,30]. In terms of the comparisons between the traditional SLR model and machine learning models, the SLR model underperformed in this study, which is different from the previous comparisons [10]. The controversy over machine learning approaches has existed for a long time. It may depend on the target air pollutants and the collecting method of monitoring data [13].

Different model performances were found for PLSU, RFU, and SLRU models developed in urban areas with less observational data involved in model development, as shown in Tables S2 and S3 in Supplementary Information. Compared with the models developed in the whole city area, the PLSU models had better performance for both NO_2_ (LOOCV R^2^_mse_: 0.83~0.96) and PM_2.5_ (LOOCV R^2^_mse_: 0.74~0.82), while RFU and SLRU models had opposite performances. The increased performance of these PLSU models might be due to overfitting. The RFU and SLRU models were sensitive to the number of observational monitoring sites. We also found worse model performances from 2018 to 2020 than those from 2015 to 2017, when observational data were missing from 2018 at the monitoring site in the western mountain area. It indicated that the monitoring sites with various observational levels were necessary for model development in the urban area [28].

**Figure 6:**
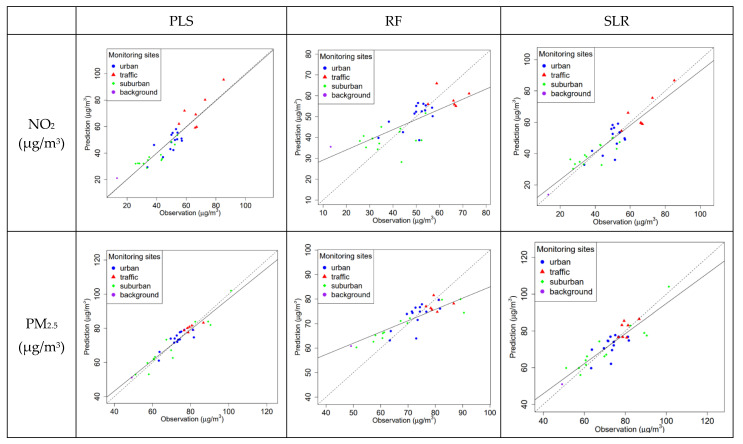
The average observations and LOOCV predictions of the three models for NO_2_ and PM_2.5_ from 2015 to 2020. Dashed lines denote the 1:1 line, and solid lines represent linear regression lines.

Figure 6 shows the scatter plots of the long-term mean observations and LOOCV predictions of the PLS, RF, and SLR mdels for NO_2_ and PM_2.5_. For NO_2_, the PLS (LOOCV R^2^_mse_: 0.78) and SLR (LOOCV R^2^_mse_: 0.76) models performed better than the RF models (LOOCV R^2^_mse_: 0.57). The RF model was overestimated at the background site. Similar results were found for PM_2.5_ models, in which the LOOCV R^2^_mse_ of PLS, RF, and SLR models were 0.80, 0.51, and 0.54, respectively. The PM_2.5_ RF model performed worse at the background site than those at other types of monitoring sites.

### 3.3. Prediction in the Urban Area

**Figure 7:**
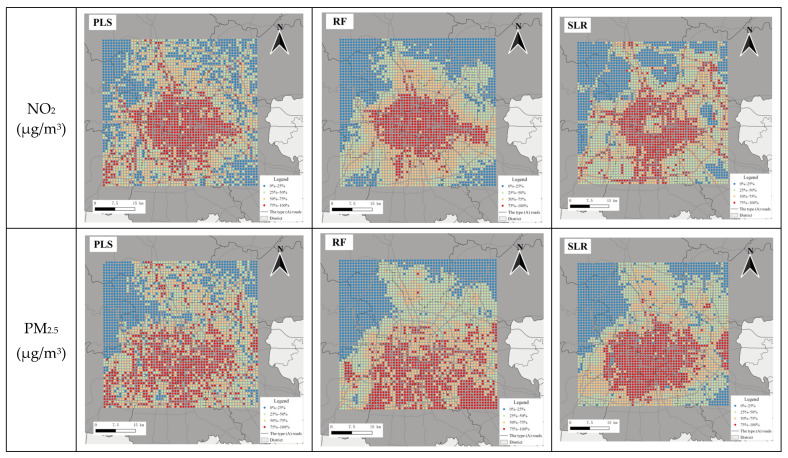
Maps of the long-term mean NO_2_ and PM_2.5_ predictions from 2015 to 2020.

A square covering the 6th ring road in Beijing was picked to show the predictions around urban areas. It was divided into 3301 grids at a 1 km spatial scale. These grids are categorized according to the quartile distributions of model predictions, as shown in Figure 7. For NO_2_, the grids with high predictions were clustered in the central urban area. In addition, the hotspots of the PLS and SLR model predictions were highlighted across the road network, while the grids with high RF predictions were aggregated (Figure 7). Regarding PM_2.5_, noticeable spatial differences were found across the three model predictions. The hotspots of the PLS model predictions were sparsely distributed, while the RF predictions were highlighted in the southern part of Beijing. In comparison, the SLR model predictions were shown with clear spatial clusters. The contrast among the three PM_2.5_ model predictions arises from the difference in the selection of geographical variables as primary predictors and the variations in the significance of predictors during model development. Figures S4 and S5 in Supplementary Information show the correlation coefficient between the three models. For LTM, the correlation coefficients of NO_2_ models among the three approaches were 0.72~0.83, and it was 0.81~0.85 in PM_2.5_ models. The correlation coefficient between PLS and SLR was the lowest regardless of whether in either NO_2_ models or PM_2.5_ models. The lower correlation coefficients of SLR with the other two models may be related to the fact that the variables were screened in SLR while the other two modeling methods were not.

**Figure 8:**
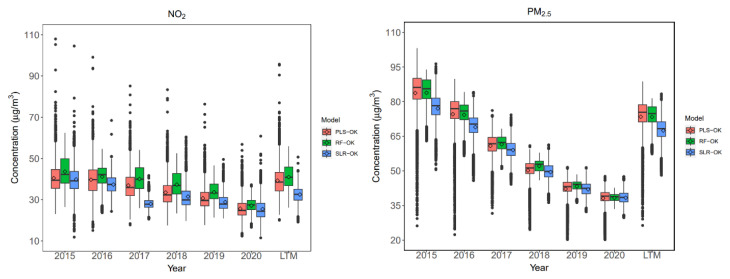
Distributions of annual averages and long-term means (LTM) from 2015 to 2020 of NO_2_ and PM_2.5_ predicted by three models. Each box’s upper, middle, and lower lines denote 75%, 50%, and 25% of the concentration. The dots represent the predictions that are higher or lower than 1.5 times the IQR from the median. The point in the box represents the means of the predictions.

Since the LUR-OK model performed better than the LUR models in LTM predictions, we focused on using the LUR-OK model to make predictions for population-weighted exposure estimates. Figure 8 depicts the box plots of the spatial predictions at a 1 km spatial scale (shown as grids in Figure 7) for NO_2_ and PM_2.5_ in the urban area. Regarding the annual predictions, a noticeable decline was found for PM_2.5_ year by year. It is expected that the air quality level in Beijing has become better in recent years [37]. Meanwhile, regarding NO_2_, the decline in annual mean concentrations was almost flat from 2015 to 2017, especially for PLS-OK and RF-OK predictions. Comparing the predictions among different models, the PLS-OK and RF-OK models had comparable median predictions. In contrast, SLR had relatively low median predictions, especially for annual mean NO_2_ prediction in 2017 (34.98 μg/m^3^) and NO_2_ and PM_2.5_ LTM predictions (NO_2_: 37.58 μg/m^3^; PM_2.5_: 71.43 μg/m^3^). The NO_2_ predictions obtained a more considerable divergence across the three models with the coefficient of variation (COV) of 11.85~19.41 for three NO_2_ models, compared with the PM_2.5_ predictions among the three models with the COV of 7.57 ~11.88. It indicated that the NO_2_ models were more sensitive to the predictors derived from the geographic variables than the PM_2.5_ models.

### 3.4. Population-Weighted Exposure Estimates

The population-weighted exposure estimates (PEE) in urban areas from 2015 to 2020 were predicted using the PLS-OK, RF-OK, and SLR-OK models. The long-term mean values of PEE in the urban area from 2015 to 2020 were calculated based on the model predictions and population density across the 1 km grids [38]. The total population in the prediction area was 15.94 million, accounting for about 76% of the entire population in Beijing, ranging from 211 to 32,097 persons/km^2^ across the grids in the urban area. The misclassification of PEE in quartiles between the three models was estimated personally.

**Figure 9:**
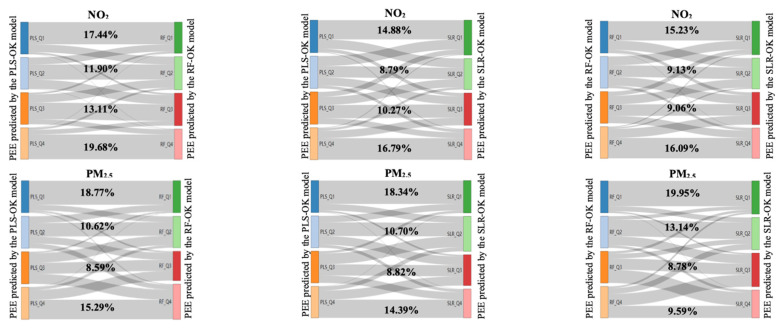
The misclassification of the PEE in quartiles between the PLS-OK, RF-OK, and SLR-OK models for NO_2_ and PM_2.5_. The four classifications from top to bottom denote the ranges of 0~25%, 25~50%, 50~75%, and 75~100% of PEE. The numbers indicate the non-misclassified percentage in quartiles.

Figure 9 depicts the misclassification of PEE in quartiles between the PLS-OK, RF-OK, and SLR-OK models. Tables S4 and S5 in Supplementary Information summarizes the percentage of misclassification in quartiles for NO_2_ and PM_2.5_. For NO_2_, the total misclassification of particular PEE between the PLS-OK and RF-OK models was 37.87%, 49.27% between PLS-OK and RF-OK models, and 50.49% between RF-OK and SLR-OK models. The contrast between PLS-OK and RF-OK models was smaller than the other two pairs. Compared with the PEE predicted by the PLS-OK model, 19.16% of them indicated by the RF-OK model were overestimated, and 18.72% were underestimated. Overestimation and underestimation between the PEE predicted using the PLS-OK and SLR-OK models were 23.25% and 26.03%, respectively. Most of the misclassifications happened across the adjacent quartiles. For comparison between the PLS-OK and RF-OK models, only 4.74% of the PEE was misclassified into the upper or lower quartiles that were not adjacent. This number was about 10% for other pairs of comparisons. It indicated that the PEE of NO_2_ predicted by PLS-OK and RF-OK models obtained similar results, which resulted in the least misclassifications of the PEE in quartiles across the three models. Regarding the PEE of PM_2.5_, the misclassification in quartiles was comparable between the three models.

### 3.5. Strengths and Limitations

This study compared three geostatistical model performances in an air pollutant exposure assessment study in a metropolitan city in China. The findings have a broad implication for environmental studies. First, the three approaches chosen for model development represent a variety of advanced and traditional, complicated, and simple geostatistical modeling methods. The usage of each modeling approach is distinctive. The SLR models outperformed the RF models. However, compared with the PLS model predictions, the RF model predictions had fewer misclassifications than the SLR predictions. Second, this study focused on spatial model comparisons, which may provide a reference for spatiotemporal modeling studies. For primary pollutants, like NO_2_, the spatial distribution of air pollutants highly relies on local features related to emission sources. A comprehensive array of geographic variables could influence the model performance at a fine spatial scale. The PLS approach has such potential for exposure assessment on directly emitted air pollutants [39]. Third, the interpretability of machine learning models is challenging. Although its usage is potentially limited because of its black box instinct, it possesses the capability for utilization in an urban area abundant with spatially dense observational data on the basis of RF model performance in this study.

There were also some limitations in this study. First, there is a lack of additional data for model development and out-of-sample validation. In this study, the observational data used for model development was derived from the national and regional monitoring network. Increasing the abundance of spatial data would enhance the accuracy and stability of the model [40]. Second, the spatial models might be overestimated as less spatially rich observational data were involved in model development. To make use of the limited spatial information from these relatively sparse monitoring sites, an out-of-sample CV was not used in this study. To address these limitations, we plan to conduct mobile monitoring in the next step of our study. In addition, the inclusion of health outcomes for analyzing the impact of exposure bias caused by choosing different exposure models will be considered.

## 4. Conclusions

Using three geostatistical modeling approaches, we developed spatial models for NO_2_ and PM_2.5_ in Beijing. After evaluating the model performances with LOOCV, we found that the PLS model exhibited the best performance among the three models. A hybrid model framework, which used OK to further explain the residuals, could improve the model performance. We compared the model performance by making predictions at a 1 km spatial scale in the urban area. The misclassification of population-weighted exposure estimates in quartiles caused by using a different modeling approach was also conducted. For NO_2_, both the PLS-OK and RF-OK models showed the least misclassification in the comparisons. The PM_2.5_ models obtained more misclassification than the NO_2_ models.

## Figures and Tables

**Table 1:** The annual average concentrations of NO_2_ and PM_2.5_ from 2015 to 2020.

Pollutant	Year	No. of Sites	Missing	Annual Averages (μg/m^3^)	SD (μg/m^3^)
NO_2_	2015	35	8.79%	49.27	15.92
	2016	34	8.79%	48.16	14.93
	2017	32	10.22%	44.09	12.57
	2018	34	3.08%	42.12	12.96
	2019	34	2.86%	37.19	10.73
	2020	34	3.62%	30.30	8.72
PM_2.5_	2015	35	4.40%	82.56	13.39
	2016	35	8.79%	74.01	11.58
	2017	32	10.33%	58.60	6.59
	2018	34	3.08%	52.58	6.32
	2019	34	2.97%	43.04	5.86
	2020	34	3.85%	38.55	5.40

**Table 2:** LOOCV results of the NO_2_ models.

Year	PLS				RF				SLR		
	RMSE	R^2^_mse_	R^2^_reg_		RMSE	R^2^_mse_	R^2^_reg_		RMSE	R^2^_mse_	R^2^_reg_
2015	6.59	0.82	0.85		9.93	0.60	0.62		7.56	0.77	0.78
2016	7.64	0.73	0.78		9.90	0.55	0.57		11.46	0.39	0.44
2017	6.19	0.75	0.78		9.09	0.46	0.46		7.00	0.68	0.71
2018	6.34	0.75	0.78		8.60	0.55	0.57		7.23	0.68	0.69
2019	5.06	0.77	0.80		7.80	0.46	0.47		5.72	0.71	0.71
2020	4.55	0.72	0.77		6.79	0.37	0.38		4.81	0.69	0.70
LTM	5.66	0.78	0.81		7.93	0.57	0.59		5.97	0.76	0.76
**Year**	**PLS-OK**				**RF-OK**				**SLR-OK**		
	**RMSE**	**R^2^_mse_**	**R^2^_reg_**		**RMSE**	**R^2^_mse_**	**R^2^_reg_**		**RMSE**	**R^2^_mse_**	**R^2^_reg_**
2015	6.18	0.84	0.87		9.75	0.61	0.62		7.43	0.78	0.78
2016	7.74	0.72	0.78		10.23	0.52	0.55		11.42	0.40	0.46
2017	5.97	0.77	0.80		9.41	0.42	0.45		6.80	0.70	0.73
2018	5.77	0.80	0.82		8.22	0.59	0.59		7.06	0.69	0.72
2019	4.61	0.81	0.83		7.76	0.46	0.47		6.11	0.67	0.67
2020	4.53	0.72	0.77		7.67	0.20	0.29		5.38	0.61	0.64
LTM	5.16	0.82	0.84		7.92	0.57	0.58		5.73	0.78	0.78

**Table 3:** LOOCV results of the PM_2.5_ models.

Year	PLS				RF				SLR		
	RMSE	R^2^_mse_	R^2^_reg_		RMSE	R^2^_mse_	R^2^_reg_		RMSE	R^2^_mse_	R^2^_reg_
2015	4.39	0.89	0.89		7.88	0.64	0.73		7.64	0.66	0.68
2016	4.07	0.87	0.87		6.90	0.64	0.73		7.02	0.62	0.63
2017	2.73	0.82	0.82		4.24	0.57	0.62		5.30	0.33	0.42
2018	2.33	0.86	0.86		4.80	0.41	0.47		4.17	0.55	0.56
2019	2.13	0.86	0.86		4.02	0.51	0.57		3.75	0.58	0.60
2020	2.21	0.83	0.83		4.21	0.37	0.40		3.43	0.58	0.59
LTM	3.51	0.80	0.80		5.51	0.51	0.59		5.33	0.54	0.54
**Year**	**PLS-OK**				**RF-OK**				**SLR-OK**		
	**RMSE**	**R^2^_mse_**	**R^2^_reg_**		**RMSE**	**R^2^_mse_**	**R^2^_reg_**		**RMSE**	**R^2^_mse_**	**R^2^_reg_**
2015	4.26	0.90	0.90		5.87	0.80	0.80		7.64	0.66	0.70
2016	4.25	0.86	0.87		5.51	0.77	0.77		7.05	0.62	0.67
2017	2.64	0.83	0.84		3.03	0.78	0.78		5.64	0.24	0.43
2018	3.45	0.69	0.72		3.99	0.59	0.59		3.90	0.61	0.62
2019	2.10	0.87	0.87		3.37	0.66	0.67		3.38	0.66	0.69
2020	2.31	0.81	0.82		4.00	0.43	0.45		3.29	0.62	0.63
LTM	3.38	0.81	0.82		4.42	0.68	0.69		5.11	0.58	0.60

## Data Availability

In this study, the observational data of air pollutants are available online. Most of the GIS features were obtained from open sources. The geographic variables are unavailable due to privacy or ethical restrictions.

## Supplementary Materials

The following supporting information can be downloaded at: https://www.mdpi.com/article/10.3390/toxics12030197/s1, Figure S1: Correlation coefficients between the first PLS score and the corresponding geographic variables in PLSU; Figure S2: The geographic variables with the top ten IncMSE values in RFU results; Figure S3: The coefficients of the variables selected by the SLRU; Figure S4: The correlation coefficients of NO_2_ models among the three approaches; Figure S5: The correlation coefficients of PM_2.5_ models among the three approaches; Table S1: Details of the geographic variables; Table S2: LOOCV results of the NO_2_ LURU models; Table S3: LOOCV results of the PM_2.5_ LURU models; Table S4: The NO_2_ misclassification between LUR models; Table S5: Quartile distribution of the PM_2.5_ misclassification between LUR models.

## References

[1] Hoek G., Beelen R., de Hoogh K., Vienneau D., Gulliver J., Fischer P., Briggs D. (2008). A review of land-use regression models to assess spatial variation of outdoor air pollution. Atmos. Environ..

[2] Brauer M., Lencar C., Tamburic L., Koehoorn M., Demers P., Karr C. (2008). A cohort study of traffic-related air pollution impacts on birth outcomes. Environ. Health Perspect..

[3] Miller K.A., Sullivan J.H. (2007). Long-Term Exposure to Air Pollution and Incidence of Cardiovascular Events in Women. N. Engl. J. Med..

[4] Shaffer R.M., Blanco M.N., Li G., Adar S.D., Carone M., Szpiro A.A., Kaufman J.D., Larson T.V., Larson E.B., Crane P.K. (2021). Fine particulate matter and dementia incidence in the adult changes in thought study. Environ. Health Perspect..

[5] Batterman S., Chambliss S., Isakov V. (2014). Spatial resolution requirements for traffic-related air pollutant exposure evaluations. Atmos. Environ..

[6] Steinle S., Reis S., Sabel C.E. (2013). Quantifying human exposure to air pollution—Moving from static monitoring to spatio-temporally resolved personal exposure assessment. Sci. Total Environ..

[7] Xiao Q.Y., Wang Y.J., Chang H.H., Meng X., Geng G.N., Lyapustin A., Liu Y. (2017). Full-coverage high-resolution daily PM2.5 estimation using MAIAC AOD in the Yangtze River Delta of China. Remote Sens. Environ..

[8] Kaufman J.D., Adar S.D., Barr R.G., Budoff M., Burke G.L., Curl C.L., Daviglus M.L., Roux A.V.D., Gassett A.J., Jacobs D.R. (2016). Association between air pollution and coronary artery calcification within six metropolitan areas in the USA (the Multi-Ethnic Study of Atherosclerosis and Air Pollution): A longitudinal cohort study. Lancet.

[9] Raaschou-Nielsen O., Andersen Z.J., Beelen R., Samoli E., Stafoggia M., Weinmayr G., Hoffmann B., Fischer P., Nieuwenhuijsen M.J., Brunekreef B. (2013). Air pollution and lung cancer incidence in 17 European cohorts: Prospective analyses from the European Study of Cohorts for Air Pollution Effects (ESCAPE). Lancet Oncol..

[10] Chen J., de Hoogh K., Gulliver J., Hoffmann B., Hertel O., Ketzel M., Bauwelinck M., van Donkelaar A., Hvidtfeldt U.A., Katsouyanni K. (2019). A comparison of linear regression, regularization, and machine learning algorithms to develop Europe-wide spatial models of fine particles and nitrogen dioxide. Environ. Int..

[11] Xu J., Yang W., Bai Z., Zhang R., Zheng J., Wang M., Zhu T. (2022). Modeling spatial variation of gaseous air pollutants and particulate matters in a Metropolitan area using mobile monitoring data. Environ. Res..

[12] Mercer L.D., Szpiro A.A., Sheppard L., Lindstroem J., Adar S.D., Allen R.W., Avol E.L., Oron A.P., Larson T., Liu L.J.S. (2011). Comparing universal kriging and land-use regression for predicting concentrations of gaseous oxides of nitrogen (NOx) for the Multi-Ethnic Study of Atherosclerosis and Air Pollution (MESA Air). Atmos. Environ..

[13] Kerckhoffs J., Hoek G., Portengen L., Brunekreef B., Vermeulen R.C.H. (2019). Performance of Prediction Algorithms for Modeling Outdoor Air Pollution Spatial Surfaces. Environ. Sci. Technol..

[14] Wang M., Brunekreef B., Gehring U., Szpiro A., Hoek G., Beelen R. (2016). A New Technique for Evaluating Land-use Regression Models and Their Impact on Health Effect Estimates. Epidemiology.

[15] Xu J., Yang Z., Han B., Yang W., Duan Y., Fu Q., Bai Z. (2022). A unified empirical modeling approach for particulate matter and NO_2_ in a coastal city in China. Chemosphere.

[16] Keller J.P., Olives C., Kim S.Y., Sheppard L., Sampson P.D., Szpiro A.A., Oron A.P., Lindstrom J., Vedal S., Kaufman J.D. (2015). A Unified Spatiotemporal Modeling Approach for Predicting Concentrations of Multiple Air Pollutants in the Multi-Ethnic Study of Atherosclerosis and Air Pollution. Environ. Health Perspect..

[17] de Hoogh K., Korek M., Vienneau D., Keuken M., Kukkonen J., Nieuwenhuijsen M.J., Badaloni C., Beelen R., Bolignano A., Cesaroni G. (2014). Comparing land use regression and dispersion modelling to assess residential exposure to ambient air pollution for epidemiological studies. Environ. Int..

[18] The People’s Government of Beijing Municipality. https://www.beijing.gov.cn.

[19] Feng L., Liao W. (2016). Legislation, plans, and policies for prevention and control of air pollution in China: Achievements, challenges, and improvements. J. Clean. Prod..

[20] WHO (2021). WHO Global Air Quality Guidelines: Particulate Matter (PM2.5 and PM10), Ozone, Nitrogen Dioxide, Sulfur Dioxide and Carbon Monoxide.

[21] Zhao S., Yu Y., Yin D., He J., Liu N., Qu J., Xiao J. (2016). Annual and diurnal variations of gaseous and particulate pollutants in 31 provincial capital cities based on in situ air quality monitoring data from China National Environmental Monitoring Center. Environ. Int..

[22] Araki S., Shima M., Yamamoto K. (2018). Spatiotemporal land use random forest model for estimating metropolitan NO_2_ exposure in Japan. Sci. Total Environ..

[23] Wu C., Fang C., Wu X., Zhu G. (2019). Health-Risk Assessment of Arsenic and Groundwater Quality Classification Using Random Forest in the Yanchi Region of Northwest China. Expo. Health.

[24] Xu J., Yang W., Han B., Wang M., Wang Z., Zhao Z., Bai Z., Vedal S. (2019). An advanced spatio-temporal model for particulate matter and gaseous pollutants in Beijing, China. Atmos. Environ..

[25] Genuer R., Poggi J.M., Tuleau-Malot C. (2010). Variable selection using Random Forests. Pattern Recognit. Lett..

[26] Beelen R., Hoek G., Vienneau D., Eeftens M., Dimakopoulou K., Pedeli X., Tsai M.Y., Kunzli N., Schikowski T., Marcon A. (2013). Development of NO_2_ and NO_x_ land use regression models for estimating air pollution exposure in 36 study areas in Europe—The ESCAPE project. Atmos. Environ..

[27] de Hoogh K., Gulliver J., van Donkelaar A., Martin R.V., Marshall J.D., Bechle M.J., Cesaroni G., Cirach Pradas M., Dedele A., Eeftens M. (2016). Development of West-European PM_2.5_ and NO_2_ land use regression models incorporating satellite-derived and chemical transport modelling data. Environ. Res..

[28] Eeftens M., Tsai M.Y., Ampe C., Anwander B., Beelen R., Bellander T., Cesaroni G., Cirach M., Cyrys J., de Hoogh K. (2012). Spatial variation of PM_2.5_, PM_10_, PM_2.5_ absorbance and PM coarse concentrations between and within 20 European study areas and the relationship with NO_2_—Results of the ESCAPE project. Atmos. Environ..

[29] Lu T., Marshall J.D., Zhang W., Hystad P., Kim S.-Y., Bechle M.J., Demuzere M., Hankey S. (2021). National Empirical Models of Air Pollution Using Microscale Measures of the Urban Environment. Environ. Sci. Technol..

[30] Sampson P.D., Richards M., Szpiro A.A., Bergen S., Sheppard L., Larson T.V., Kaufman J.D. (2013). A regionalized national universal kriging model using Partial Least Squares regression for estimating annual PM_2.5_ concentrations in epidemiology. Atmos. Environ..

[31] Kim S.-Y., Bechle M., Hankey S., Sheppard L., Szpiro A.A., Marshall J.D. (2020). Concentrations of criteria pollutants in the contiguous U.S., 1979–2015: Role of prediction model parsimony in integrated empirical geographic regression. PLoS ONE.

[32] Wang L., Liu Z., Sun Y., Ji D., Wang Y. (2015). Long-range transport and regional sources of PM_2.5_ in Beijing based on long-term observations from 2005 to 2010. Atmos. Res..

[33] Zhang L., Tian X., Zhao Y., Liu L., Li Z., Tao L., Wang X., Guo X., Luo Y. (2021). Application of nonlinear land use regression models for ambient air pollutants and air quality index. Atmos. Pollut. Res..

[34] Dong J., Cai X., Tian L., Chen F., Xu Q., Li T., Chen X. (2023). Satellite-based estimates of daily NO_2_ exposure in urban agglomerations of China and application to spatio-temporal characteristics of hotspots. Atmos. Environ..

[35] Lyu Y., Kirwa K., Young M., Liu Y., Liu J., Hao S., Li R., Xu D., Kaufman J.D. (2022). A high-resolution computationally-efficient spatiotemporal model for estimating daily PM_2.5_ concentrations in Beijing, China. Atmos. Environ..

[36] Shi T., Dirienzo N., Requia W.J., Hatzopoulou M., Adams M.D. (2020). Neighbourhood scale nitrogen dioxide land use regression modelling with regression kriging in an urban transportation corridor. Atmos. Environ..

[37] Xu J., Zhang Z., Zhao X., Cheng S. (2023). Downward trend of NO_2_ in the urban areas of Beijing-Tianjin-Hebei region from 2014 to 2020: Comparison of satellite retrievals, ground observations, and emission inventories. Atmos. Environ..

[38] National Bureau of Statistics. https://www.stats.gov.cn/english/.

[39] Ma X., Zou B., Deng J., Gao J., Longley I., Xiao S., Guo B., Wu Y., Xu T., Xu X. (2024). A Comprehensive Review of the Development of Land Use Regression Approaches for Modeling Spatiotemporal Variations of Ambient Air Pollution: A Perspective from 2011 to 2023. Environ. Int..

[40] Basagana X., Rivera M., Aguilera I., Agis D., Bouso L., Elosua R., Foraster M., de Nazelle A., Nieuwenhuijsen M., Vila J. (2012). Effect of the number of measurement sites on land use regression models in estimating local air pollution. Atmos. Environ..

