# Supplementary material for: Comparison of Population-Weighted Exposure Estimates of Air Pollutants Based on Multiple Geostatistical Models in Beijing, China
Source: Toxics. 2024 Mar 1;12(3):197. doi: 10.3390/toxics12030197 (PMC10976140; doi:10.3390/toxics12030197)
Supplement: Supplementary file 1 [file toxics-12-00197-s001.zip › toxics-2863655-supplementary.pdf]

Supporting Information

Comparison of population exposure estimates using multiple geostatistical models in Beijing, China

Yinghan Wu[a], Jia Xu*[a], Ziqi Liu[a,b], Bin Han[a], Wen Yang*[a], Zhipeng Bai[a,b].

a. State Key Laboratory of Environmental Criteria and Risk Assessment, Chinese Research Academy of Environmental Sciences, Beijing 10012, China

b. Environmental & Occupational Health Sciences, School of Public Health, University of Washington, Seattle, WA 98105, United States

Contents

1 Geographic variables......2
Table S1 Details of the geographic variables......2
2 Model development......5
2.1 PLSU Results......5
Figure S1. Correlation coefficients between the first PLS score and the corresponding geographic variables in PLSU......5
2.2 RFU Results......5
Figure S2. The geographic variables with the top ten IncMSE values in RFU results......6
2.3 SLRU Results......6
Figure S3. The coefficients of the variables selected by the SLRU......6
3 Model Performance......6
Table S2 LOOCV results of the NO_2 LURU models......6
Table S3 LOOCV results of the $PM_{2.5}$ LURU models......7
4 Correlation relationships for LUR model predictions......7
Figure S4. The correlation coefficients of NO_2 models among the three approaches......8
Figure S5. The correlation coefficients of $PM_{2.5}$ models among the three approaches......8
5 Population exposure estimates......9
Table S4 the NO_2 misclassification between LUR models......9
Table S5 Quartile distribution of the $PM_{2.5}$ misclassification between LUR models......10

1 Geographic variables

Table S1. Details of the geographic variables

Category	Variable	Variable name in model	Buffer radii (m)[a]	Data source
Population	Population density (National Bureau of Statistics of China)	Pop	500,1000,1500,2000,2500,3000,5000,10000,15000	Resource and Environment Science and Data Center (http://www.resdc.cn/)
Topography	Elevation (Chinese Academy of Sciences)	Elevation	NA	Resource and Environment Science and Data Center (http://www.resdc.cn/)
Traffic	Distance to the nearest major roads	Log_m_to_road_a/b/c[*]	NA	OpenStreetMap(http://www.openstreetmap.org)
	Distance to the nearest bus routes	Log_m_to_bus		
	Distance to the nearest major intersections	Log_m_to_inter_aa/ab[**]		
	Sum of major roads length within a buffer	Ll_road_a/b/c	50,100,150,200,300,400,500,750,1000,1500,3000,5000	
	Sum of bus route length within a buffer	Ll_bus		
Distance to feature	Airport (large airport/other airport)	Log_m_to_airp_large/other	NA	OpenStreetMap(http://www.openstreetmap.org)
	Railway	Log_m_to_railway		
	Railyard	Log_m_to_rail yard		
Emission	Count of bus stops	Poi_bus_stop	250,500,1000,1500,2000,2500,3000,5000,7500,10000,15000	OpenStreetMap(http://www.openstreetmap.org)
	Count of gas station	Poi_gas_station		
	Count of industry	Poi_industry		
	Count of temple	Poi_temple		

	Count of restaurant	Poi_restaurant		
	Count of parking	Poi_parking		
Land-use (percent of land use category)	Cropland	LU: cropland(lu_cropland)	50,100,150,300,400,500,750,1000,1500,3000	Global land cover 2017 (http://data.ess.tsinghua.edu.cn)
	Forest	LU: forest(lu_forest)		
	Grassland	LU: grassland(lu_grassland)		
	Shrubland	LU: shrubland(lu_shrubland)		
	Wetland	LU: wetland(lu_wetland)		
	Water	LU: water(lu_water)		
	Tundra	LU: tundra(lu_tundra)		
	Impervious surface	LU: impervious(lu_impervious)		
NDVI	the 25th percentile of 2010 Normalized Difference Vegetation Index (NDVI) values	NDVI: q25(ndvi_q25)	250,500,1000,2500,5000,7500,10000	Resource and Environment Science and Data Center (http://www.resdc.cn/)
	the 50th percentile of 2010 NDVI values	NDVI: q50(ndvi_q50)		
	the 75th percentile of 2010 NDVI values	NDVI: q75(ndvi_q75)		
	the 50th percentile of 2010 NDVI values, January through March and October through December	NDVI: winter(ndvi_winter)		
	the 50th percentile of 2010 NDVI values, April through September	NDVI: summer(ndvi_summer)		
Longitude and latitude variables	Latitude in CGCS2000 / 3-degree Gauss-Kruger CM 117E(EPSG:4548)	Lambert_x	NA	NASA-EARTHDATA (http://lpdaac.usgs.gov/products/mcd19a2v006)

	Longitude in CGCS2000 / 3-degree Gauss-Kruger CM 117E(EPSG:4548)	Lambert_y		

*Road a are motorway and trunk which fclass is motorway and trunk in OpenStreetMap. Road b is primary way which fclass is primary in OpenStreetMap. Road c is secondary road which fclass is secondary in OpenStreetMap.

**Inter_aa is intersection between road a and road a. Inter_ab is intersection between road a and road b.

2 Model development

2.1 PLSU Results

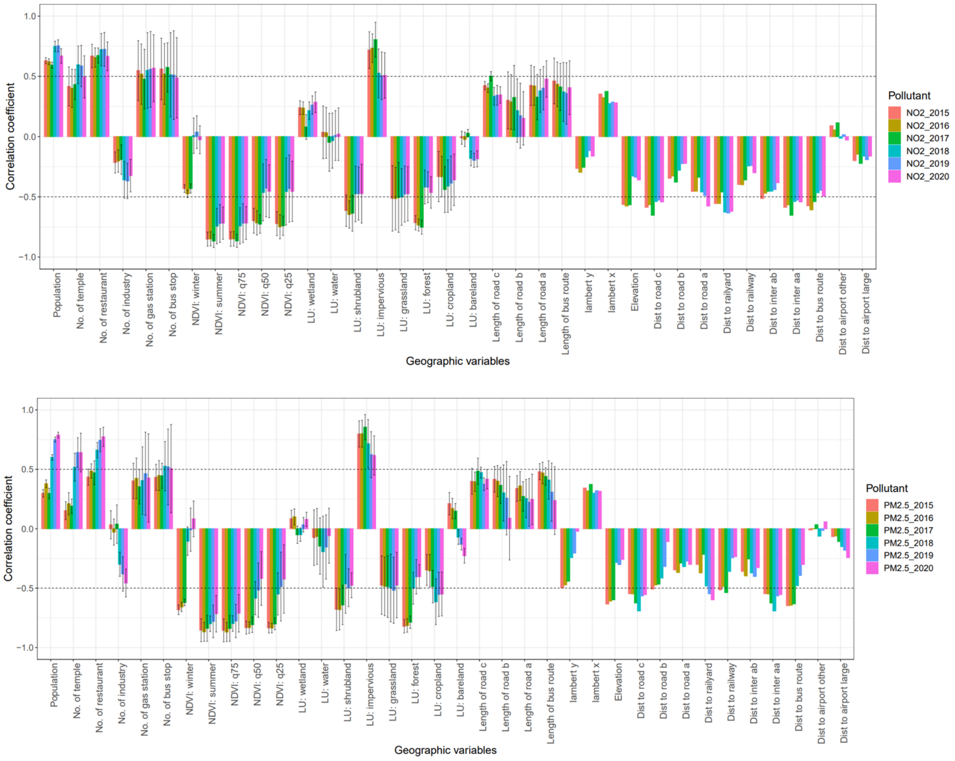

Figure S1. Correlation coefficients between the first PLS score and the corresponding geographic variables in PLSU.

2.2 RFU Results

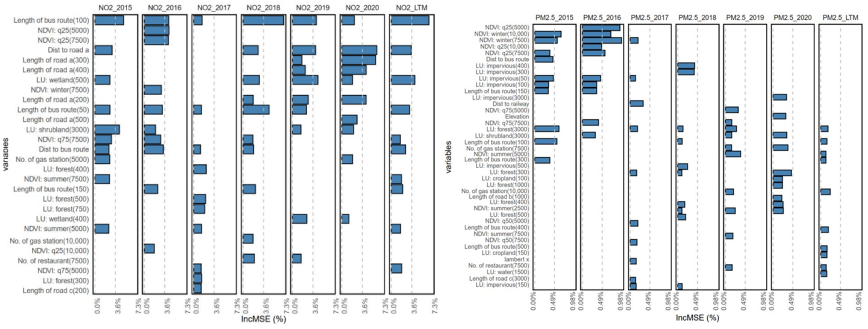

Figure S2. The geographic variables with the top ten IncMSE values in RFU results

2.3 SLRU Results

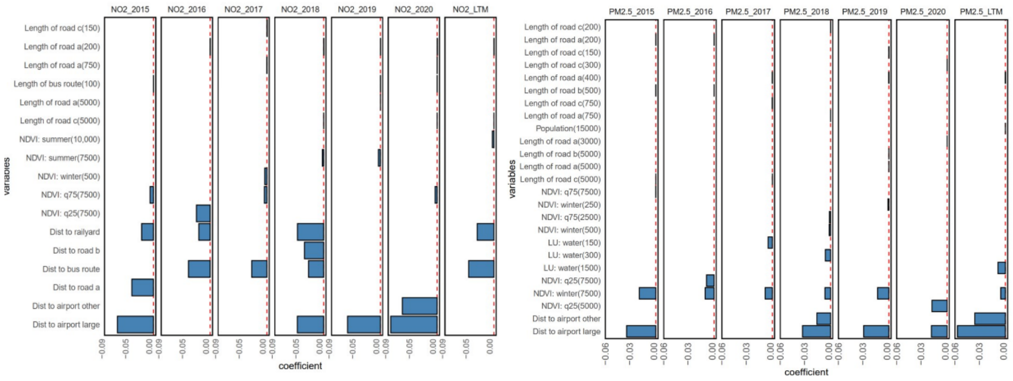

Figure S3. The coefficients of the variables selected by the SLRU

3 Model Performance

Table S2. LOOCV results of the NO_2 LURU models

Year	PLSU			RFU			SLRU		
	RMSE	R^2_{mse}	R^2_{reg}	RMSE	R^2_{mse}	R^2_{reg}	RMSE	R^2_{mse}	R^2_{reg}
2015	3.75	0.92	0.92	10.37	0.35	0.42	6.14	0.77	0.77
2016	3.49	0.89	0.89	9.05	0.25	0.26	8.91	0.27	0.35
2017	3.52	0.83	0.83	7.36	0.24	0.24	5.05	0.64	0.66
2018	3.19	0.90	0.90	8.55	0.27	0.32	5.24	0.72	0.73
2019	1.87	0.95	0.95	7.08	0.28	0.32	4.58	0.70	0.72
2020	1.29	0.96	0.96	4.71	0.46	0.61	2.89	0.80	0.80
LTM	2.71	0.91	0.91	7.18	0.40	0.47	5.31	0.67	0.67

Year	PLSU-OK			RFU-OK			SLRU-OK		
	RMSE	R^2_{mse}	R^2_{reg}	RMSE	R^2_{mse}	R^2_{reg}	RMSE	R^2_{mse}	R^2_{reg}
2015	3.62	0.92	0.92	9.94	0.41	0.43	7.51	0.66	0.53
2016	3.78	0.87	0.88	9.81	0.12	0.15	7.49	0.48	0.50
2017	3.40	0.84	0.85	7.37	0.23	0.24	5.20	0.62	0.63
2018	3.26	0.89	0.89	8.29	0.31	0.35	8.39	0.29	0.41
2019	1.86	0.95	0.95	6.95	0.31	0.32	7.57	0.18	0.36
2020	1.83	0.92	0.91	4.86	0.42	0.47	4.88	0.42	0.53
LTM	2.72	0.91	0.91	6.95	0.44	0.48	5.83	0.60	0.63

Table S3. LOOCV results of the $PM_{2.5}$ LURU models

Year	PLSU			RFU			SLRU		
	RMSE	R^2_{mse}	R^2_{reg}	RMSE	R^2_{mse}	R^2_{reg}	RMSE	R^2_{mse}	R^2_{reg}
2015	2.26	0.90	0.90	6.28	0.25	0.27	5.42	0.44	0.52
2016	1.88	0.90	0.90	4.90	0.33	0.35	3.00	0.75	0.75
2017	1.71	0.74	0.74	3.09	0.15	0.16	2.79	0.31	0.40
2018	0.92	0.89	0.89	2.31	0.30	0.31	2.10	0.42	0.52
2019	1.05	0.82	0.82	2.12	0.27	0.27	1.70	0.53	0.55
2020	1.13	0.85	0.85	2.73	0.09	0.10	2.66	0.13	0.19
LTM	1.29	0.85	0.86	3.56	0.00	0.00	4.23	0.00	0.05
Year	PLSU-OK			RFU-OK			SLRU-OK		
	RMSE	R^2_{mse}	R^2_{reg}	RMSE	R^2_{mse}	R^2_{reg}	RMSE	R^2_{mse}	R^2_{reg}
2015	2.19	0.91	0.91	5.19	0.49	0.51	5.60	0.41	0.49
2016	1.84	0.91	0.91	4.64	0.40	0.42	3.07	0.74	0.74
2017	1.72	0.73	0.74	2.90	0.25	0.25	2.89	0.26	0.37
2018	1.03	0.86	0.86	2.54	0.15	0.17	2.14	0.40	0.51
2019	1.15	0.79	0.79	2.18	0.23	0.30	1.69	0.53	0.55
2020	1.20	0.82	0.83	3.17	0.00	0.01	2.93	0.00	0.10
LTM	1.32	0.85	0.85	3.92	0.00	0.00	4.28	0.00	0.03

4 Correlation relationships for LUR model predictions

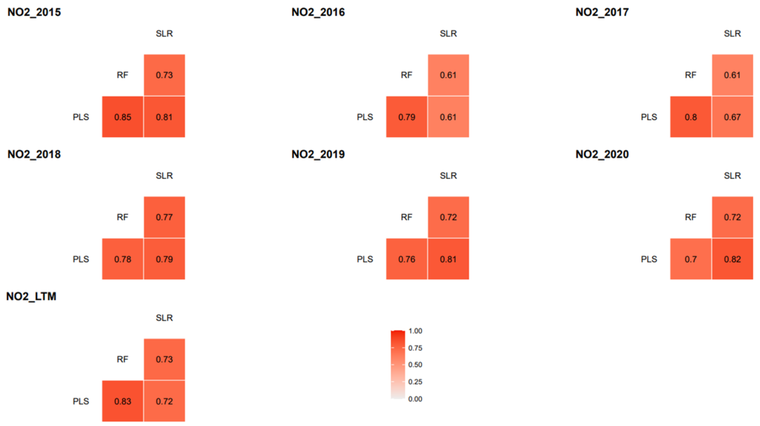

(a) LUR models

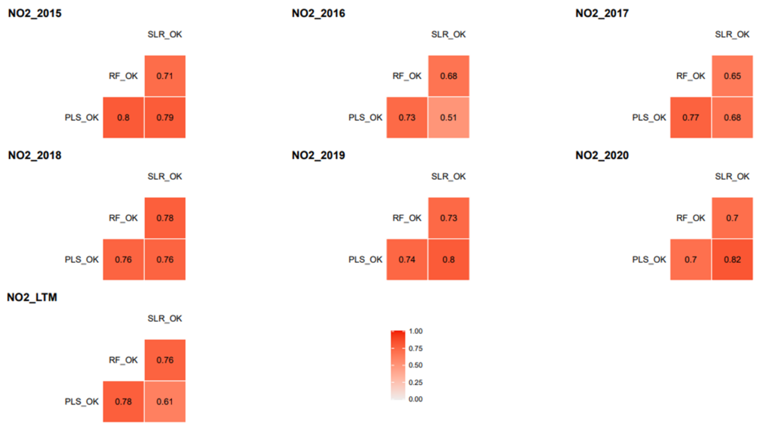

(b) LUR+OK models

Figure S4. The correlation coefficients of NO_2 models among the three approaches

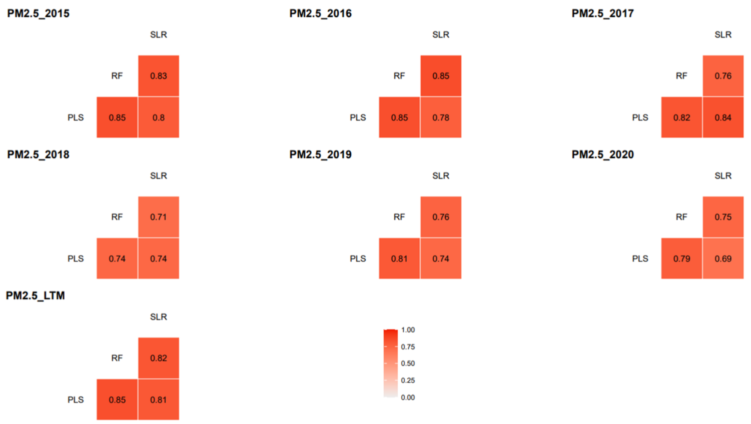

(a) LUR models

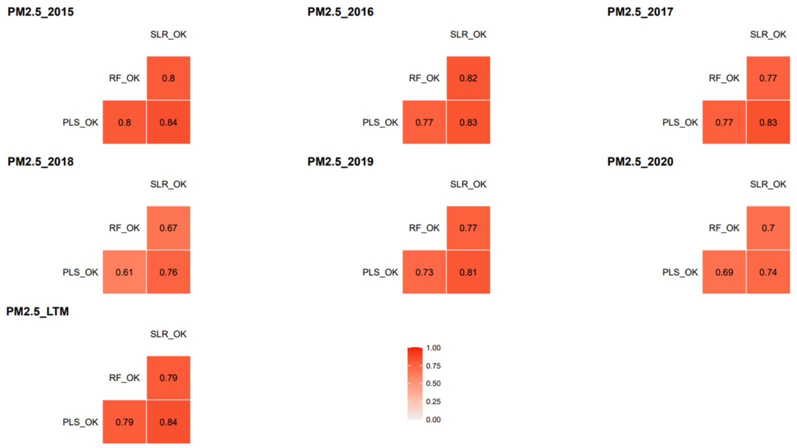

(b) LUR+OK models

Figure S5. The correlation coefficients of $PM_{2.5}$ models among the three approaches

5 Population exposure estimates

Table S4. the NO_2 misclassification between LUR models

source	target	value	Proportion	source	target	value	Proportion	source	target	value	Proportion
PLS_Q1	RF_Q1	3760801	17.44%	PLS_Q1	SLR_Q1	3209409	14.88%	RF_Q1	SLR_Q1	3284007	15.23%
PLS_Q1	RF_Q2	1327762	6.16%	PLS_Q1	SLR_Q2	1528142	7.09%	RF_Q1	SLR_Q2	1582834	7.34%
PLS_Q1	RF_Q3	317288.9	1.47%	PLS_Q1	SLR_Q3	558239.9	2.59%	RF_Q1	SLR_Q3	515185.9	2.39%
PLS_Q1	RF_Q4	0	0.00%	PLS_Q1	SLR_Q4	110060.9	0.51%	RF_Q1	SLR_Q4	23825.02	0.11%
PLS_Q2	RF_Q1	1198096	5.56%	PLS_Q2	SLR_Q1	1656884	7.68%	RF_Q2	SLR_Q1	1518888	7.04%
PLS_Q2	RF_Q2	2565447	11.90%	PLS_Q2	SLR_Q2	1894599	8.79%	RF_Q2	SLR_Q2	1969168	9.13%
PLS_Q2	RF_Q3	1566259	7.26%	PLS_Q2	SLR_Q3	1349503	6.26%	RF_Q2	SLR_Q3	1487255	6.90%
PLS_Q2	RF_Q4	56109.36	0.26%	PLS_Q2	SLR_Q4	484925.6	2.25%	RF_Q2	SLR_Q4	410600.1	1.90%
PLS_Q3	RF_Q1	307918.6	1.43%	PLS_Q3	SLR_Q1	831808.5	3.86%	RF_Q3	SLR_Q1	723015	3.35%
PLS_Q3	RF_Q2	1386477	6.43%	PLS_Q3	SLR_Q2	1358746	6.30%	RF_Q3	SLR_Q2	1466740	6.80%
PLS_Q3	RF_Q3	2826605	13.11%	PLS_Q3	SLR_Q3	2214494	10.27%	RF_Q3	SLR_Q3	1952956	9.06%
PLS_Q3	RF_Q4	864911	4.01%	PLS_Q3	SLR_Q4	980862.7	4.55%	RF_Q3	SLR_Q4	1243200	5.77%
PLS_Q4	RF_Q1	2308.25	0.01%	PLS_Q4	SLR_Q1	112023.8	0.52%	RF_Q4	SLR_Q1	164522.8	0.76%
PLS_Q4	RF_Q2	341353.9	1.58%	PLS_Q4	SLR_Q2	384541.1	1.78%	RF_Q4	SLR_Q2	214375.3	0.99%
PLS_Q4	RF_Q3	799204.3	3.71%	PLS_Q4	SLR_Q3	1269543	5.89%	RF_Q4	SLR_Q3	1537224	7.13%
PLS_Q4	RF_Q4	4243045	19.68%	PLS_Q4	SLR_Q4	3619803	16.79%	RF_Q4	SLR_Q4	3469789	16.09%

Table S5. Quartile distribution of the $PM_{2.5}$ misclassification between LUR models

source	target	value	Proportion	source	target	value	Proportion	source	target	value	Proportion
PLS_Q1	RF_Q1	4046977	18.77%	PLS_Q1	SLR_Q1	3955174	18.34%	RF_Q1	SLR_Q1	4301292	19.95%
PLS_Q1	RF_Q2	964879.6	4.47%	PLS_Q1	SLR_Q2	1095572	5.08%	RF_Q1	SLR_Q2	843096.1	3.91%
PLS_Q1	RF_Q3	267833.7	1.24%	PLS_Q1	SLR_Q3	290501.8	1.35%	RF_Q1	SLR_Q3	249458.6	1.16%
PLS_Q1	RF_Q4	126161.9	0.59%	PLS_Q1	SLR_Q4	64604.67	0.30%	RF_Q1	SLR_Q4	12005.1	0.06%
PLS_Q2	RF_Q1	1115621	5.17%	PLS_Q2	SLR_Q1	1096322	5.08%	RF_Q2	SLR_Q1	595865.6	2.76%
PLS_Q2	RF_Q2	2290073	10.62%	PLS_Q2	SLR_Q2	2308381	10.70%	RF_Q2	SLR_Q2	2832411	13.14%
PLS_Q2	RF_Q3	1174155	5.45%	PLS_Q2	SLR_Q3	1466328	6.80%	RF_Q2	SLR_Q3	1527460	7.08%
PLS_Q2	RF_Q4	806061.5	3.74%	PLS_Q2	SLR_Q4	514880.5	2.39%	RF_Q2	SLR_Q4	430175.1	1.99%
PLS_Q3	RF_Q1	235055.1	1.09%	PLS_Q3	SLR_Q1	392401.6	1.82%	RF_Q3	SLR_Q1	425781.8	1.97%
PLS_Q3	RF_Q2	1603270	7.44%	PLS_Q3	SLR_Q2	1839428	8.53%	RF_Q3	SLR_Q2	812405.8	3.77%
PLS_Q3	RF_Q3	1851966	8.59%	PLS_Q3	SLR_Q3	1902380	8.82%	RF_Q3	SLR_Q3	1893037	8.78%
PLS_Q3	RF_Q4	1695620	7.86%	PLS_Q3	SLR_Q4	1251702	5.80%	RF_Q3	SLR_Q4	2254686	10.46%
PLS_Q4	RF_Q1	10915.37	0.05%	PLS_Q4	SLR_Q1	24959.03	0.12%	RF_Q4	SLR_Q1	305019.9	1.41%
PLS_Q4	RF_Q2	440515.7	2.04%	PLS_Q4	SLR_Q2	695317.1	3.22%	RF_Q4	SLR_Q2	987634.5	4.58%
PLS_Q4	RF_Q3	1637751	7.59%	PLS_Q4	SLR_Q3	1561933	7.24%	RF_Q4	SLR_Q3	2024853	9.39%
PLS_Q4	RF_Q4	3296730	15.29%	PLS_Q4	SLR_Q4	3103702	14.39%	RF_Q4	SLR_Q4	2068404	9.59%
